# Mimicking Hypoxic-Ischemic Encephalopathy in a Newborn with 21q Deletion Originating from Ring Chromosome 21

**DOI:** 10.3390/children10091461

**Published:** 2023-08-27

**Authors:** Ja Un Moon, Sook Kyung Yum

**Affiliations:** 1 Department of Pediatrics, Yeouido St. Mary’s Hospital, College of Medicine, The Catholic University of Korea, Seoul 07345, Republic of Korea; 2 Department of Pediatrics, Seoul St. Mary’s Hospital, College of Medicine, The Catholic University of Korea, Seoul 06591, Republic of Korea

**Keywords:** 21q deletion, hypoxic-ischemic encephalopathy, ring chromosome, microdeletion, refractory seizure

## Abstract

Partial deletion of the long arm (q) in chromosome 21 is an extremely rare condition with various phenotypes, including microcephaly, neurodevelopmental delay, dysmorphic features, and epileptic seizures. Neonatal hypoxic-ischemic encephalopathy (HIE) is an encephalopathy associated with a hypoxic-ischemic event in the brain where seizures usually occur in the earliest days of life. Neonatal encephalopathy is a distinct entity resulting from metabolic disorders, congenital infections or genetic abnormalities that could often mimic HIE features, leading to a misdiagnosis of HIE. Here, we present a case of a newborn who was initially misdiagnosed with HIE due to HIE-like features, and eventually was diagnosed to have a de novo ring chromosome 21 with 21q microdeletion. Clinical findings, including severe hypotonia with respiratory/feeding difficulties and intractable seizures, and radiologic findings of ischemic encephalopathy were discovered. Subsequent atypical findings of the clinical presentation ultimately led to her undergoing genetic testing confirming that she had a neonatal encephalopathy with a genetic abnormality. Our case highlights the importance of identifying non-HI neonatal encephalopathy by careful and structured evaluation for current history with a clinical course and a multidisciplinary approach including genetic testing, to provide an accurate diagnosis, treat curable inherited disorders, and develop future genetic counseling.

## 1. Introduction

Partial deletion of the long arm (q) in chromosome 21 is an infrequent chromosome abnormality that occurs in less than one per million births. Most cases of 21q deletions are attributed to chromosomal abnormalities. Roberson et al. [1] found that the majority of the cases of chromosome 21q deletion were related to rearrangements including translocations, deletions and duplications involving chromosomes other than 21. However, chromosome 21q deletion in the current study occurred during the formation of ring chromosome 21. Clinical manifestations such as short stature, microcephaly, neurodevelopmental delay, dysmorphic features, skeletal and cardiac defects, and epileptic seizures have been commonly observed in chromosome 21q deletion [1,2]. Malformation of the brain (dysplasia of corpus callosum and holoprosencephaly), the most severe phenotype, has also been reported, but less frequently [3].

Neonatal hypoxic-ischemic encephalopathy (HIE) is a specific diagnosis occurring worldwide and applies only if an encephalopathy is associated with decreased oxygenation or reduced blood flow to the brain resulting in a hypoxic-ischemic event [4,5]. Early assessment using a combination of clinical variables, including neurologic status, laboratory values, and neuroimaging, plays a critical role in promptly initiating therapeutic hypothermia to mitigate the secondary brain injury caused by the hypoxic event. HIE is one of the well-known leading causes of brain injury that might cause neonatal seizures or encephalopathy during the earliest days of life [6]. Seizures usually occur in approximately 40 to 60% of the infants with HIE [5] within the first 1–2 days of birth and then subside after a few days as the condition improves. However, seizures might be clustered/prolonged or recur for weeks or years in some severe cases. 

Recent technological advances in genomics have shown that neonatal-onset seizures are not only associated with HIE, but also with neonatal encephalopathy, a distinct entity [7]. Neonatal encephalopathy could be broadly defined as a disturbed neurologic function manifesting in disturbance of consciousness, decreased spontaneous activity, abnormal tone, respiratory depression, impaired feeding, epileptic seizures, and abnormal reflexes together with abnormal brain imaging study or/and EEG [8]. However, neonatal encephalopathy due to other etiologies presenting HIE-like features could easily mask a diagnosis of HIE. 

Herein, we present a rare case of a newborn who was initially under suspicion of HIE until subsequent clinical findings led to a final diagnosis of ring chromosome 21 associated with 21q microdeletion.

## 2. Case Description

A 40^+1^-week gestation female baby was born via normal vaginal delivery at a local obstetric clinic. Birth weight was 3300 g (50th centile), length 52 cm (50th centile), and head circumference 35 cm (10–50th centile). The mother was 32 years old with parity of 1-0-0-1 and uneventful prenatal conditions. At birth, the baby was thickly meconium-stained but vigorous, resulting in Apgar scores of 9 at 1 min, then 10 at both 5 and 10 min.

At 3 h of life, she was transferred to our neonatal intensive care unit (NICU) due to grunting, tachypnea, and swirling movements of her arms. Upon clinical examination on arrival, she presented increasing respiratory distress with desaturation requiring a humidified high-flow nasal cannula. There were no dysmorphic features or other bony deformities. She was mildly hypotonic, irritable, and difficult to comfort, but had no problems in other neurologic examinations including primitive reflexes. The amplitude-integrated electroencephalogram (aEEG) showed a continuous pattern without electrographic seizure waves. However, she subsequently developed a generalized tonic seizure a few hours from admission and required ventilator support. As the aEEG presented typical status epilepticus waveforms, she was investigated and initially managed under the suspicion of HIE. Blood tests, including arterial blood gas, glucose, electrolyte levels, and screening for metabolic disorders such as ammonia and lactate, were unremarkable. Other examinations including chest X-ray, echocardiography, and urine analysis were all normal.

**Figure 1:**
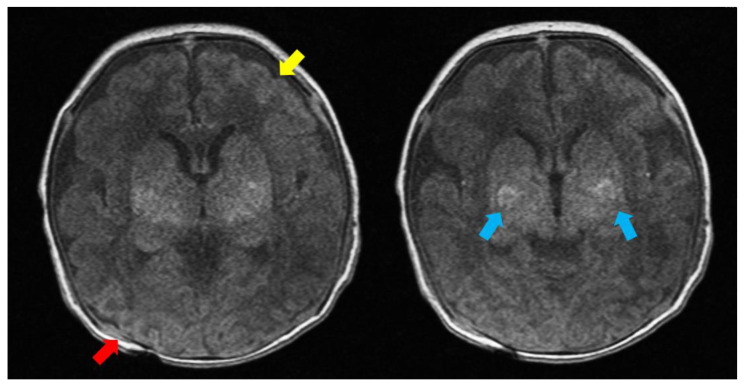
Brain MRI T1-weighted axial images on day 3 showing multifocal hypoxic-ischemic lesions in both putamen and thalami (blue arrows), right occipital (red arrow) and left frontal (yellow arrow) subcortical white matter.

According to the neonatal status epilepticus treatment, anti-seizure medication (ASM) was administrated without any delay. Although two consecutive loading doses of phenobarbital, levetiracetam as well as fosphenytoin, and additional continuous infusion of midazolam were subsequently administered intravenously, her seizures were difficult to control. The brain magnetic resonance imaging (MRI) performed on day 3 revealed multifocal hypoxic-ischemic lesions in both putamen and thalami, as well as in the right occipital and left frontal subcortical white matter (Figure 1). The initial bedside electroencephalogram (EEG) performed on day 4 showed a prolonged interval of absent cortical activity and frequent multifocal epileptiform discharges with interhemispheric asynchrony. A repeat EEG on day 7 showed a worsening background activity with two episodes of electrographic seizures. Both clinical and electrographic seizures remained despite additional multiple ASM regimens. Neither the cerebral spinal tap nor the follow-up brain MRI revealed particular findings likely responsible for the uncontrolled seizures. Although the frequency of myoclonic movements was reduced after starting valproic acid and topiramate, electrographic seizures were still found on a follow-up EEG, so clonazepam and pyridoxine were added. Apart from continued bedside physiotherapy and occupational therapy, severe generalized hypotonia and poor sucking/swallowing eventually led to feeding and respiratory problems. Short-repeated epileptic seizures remained regardless of multiple combinations of ASMs, resulting in intractable seizures. Her seizure frequency became responsive to medications over time, but not completely resolved. The current regimen includes valproic acid and clonazepam.

**Figure 2:**
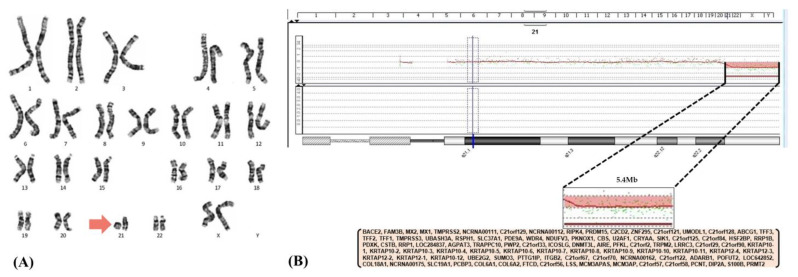
A ring chromosome 21 (**A**, red arrow) with a 5.4 Mb microdeletion (**B**) identified on chromosome analysis and microarray, respectively.

Since the clinicians deemed that she deviated from the typical clinical course of HIE, genetic evaluation was performed with the parents’ informed consent. Conventional cytogenetic investigations on lymphocytes from her peripheral blood revealed ring chromosome 21, resulting in a karyotype of 46,XX,r(21) (Figure 2A). Further array-based comparative genomic hybridization (aCGH) was conducted to detect the size and location of genetic deletion/gains which are often accompanied by chromosomal aberration. She was finally confirmed as having ring chromosome 21 with a 5.4 Mb length of deletion at the 21q22.3 (42622651_48056450) region (Figure 2B). There was no abnormality in her parents’ genetic testing, confirming a de novo chromosome 21q deletion. 

With the parents’ consent, she was discharged from the NICU with the assistance of an orogastric tube on day 56. At 10 months, she still required multiple ASMs and an orogastric tube for feeding as well as physiotherapy/occupational therapy at the rehabilitative medicine department due to severe hypotonia.

## 3. Discussion

Ring chromosome 21, first described by Lejeune in 1964 [9], is a chromosome aberration resulting from breakage and reunion at the breakpoints on the long and short arms of chromosome 21 to form a ring, resulting in the deletion of a variable amount of chromosomal segments distal to the breakpoints. Previous studies have reported that patients with ring chromosome 21 present with inconsistent phenotypes [9,10]. Its phenotype has a diverse range from almost asymptomatic to severe depending on the amount of missing genetic material in the critical region at the two ends of chromosome 21. Typical phenotypes identified in children with ring chromosome 21 include distinctive dysmorphic features, congenital anomalies, and cognitive impairment. Other phenotypes, such as microcephaly, growth retardation, heart defects, cleft lip/palate, and hematologic disorders including thrombocytopenia, have also been reported [1,10,11,12,13]. 

Chromosomal aberrations frequently identified in relation with epilepsy were involved with ring chromosomes 14, 17 and 20 [14]. However, only a few cases of ring chromosome 21 accompanied with epilepsy as well as poor description of EEG findings have been reported until now. Seizure types, including both focal and generalized onset and multifocal or generalized slow epileptiform abnormalities on EEG, have previously been described in the literatures [11,13,15,16,17,18,19]. Given that some cases representing seizures/epilepsy in ring chromosome 21 were associated with loss of 21q genetic materials [15,16], we deemed that these deleted portions could harbor potential candidate epilepsy genes. We hypothesize that these genes or genes with unclear function have been primarily responsible for the refractory seizures in the current case. Specchio et al. [16] discussed a boy with 46,XY,r(21)(p13q22.3)/45,XY,-21 karyotype and the phenotype of epilepsy, intellectual disability, and mild dysmorphic features similar to our case. This also supports potential pathogenic epileptic genes associated with chromosome 21 might be located in region 3, near to the telomere in the long arm. Among the genes located in 21q22.3, a variant in *SIK1* was rated as an important factor related with epileptic encephalopathy, and it was demonstrated in an in vitro neuronal model that the *SIK1* mutation resulted in synaptic dysregulations and reduced neurites causing epilepsy in some cases [20]. Simsek-Kiper et al. identified pathogenic variants in subunits of collagen VI (COL6A1, COL6A2 and COL6A3) involving the deletion in region 3, especially 21q22.3, which subsequently led to marked muscle weakness as well as cardiac anomaly, global developmental delay and mild dysmorphic features in a boy diagnosed with collagen VI-related muscular dystrophies [21]. Since the boy was also confirmed as having ring chromosome 21, marked muscle weakness which is consistent with severe hypotonic manifestation in our case implies that heterogenous phenotypes of 21q deletion not only depend on the deleted region (21q22.3) but they are also affected by concordant chromosomal anomalies. Partial deletion in 21q22.2–22.3 has been reported to disturb cortical development resulting in structural brain malformations, including hypoplasia of the corpus callosum, polymicrogyria, and cerebellar hypoplasia [3]. Even though our case had some overlapped genes contributing to brain morphological changes, no such structural brain malformation was confirmed by brain MRI except hypoxic ischemic lesions. This difference could be explained by multiple “unknown” genes mapped in this region regulating brain development. Moreover, Ehiling et al. demonstrated the variability in morphogenetics with genetic mutations supporting the various phenotypes in similar deletions in the same region [22]. Overall, the heterogeneity of 21q deletion attributed to the region and extent of deletion, presence of concordant chromosomal abnormalities as well as unrecognized epistatic factors indicating further advances on genetic testing in specific deletion regions, could offer an opportunity to identify potential genes and allow early therapies and interventions.

Neonates manifesting neurological abnormalities at or shortly after birth could easily lead to misdiagnosis as HIE. The underlying etiologies of non-HI neonatal encephalopathy include genetic abnormalities, metabolic disorders, vascular lesions (e.g., stroke), and congenital/early-onset infection [23]. Previous studies demonstrated that the pathogenic genetic variants contributing to neonatal encephalopathy have been accounted for between 10 and 40% [8,24]. It is important to distinguish non-HI neonatal encephalopathy from HIE in terms of the treatable inherited genetic disorder. Apart from refractory seizures, genetic abnormalities should be considered in newborns with encephalopathy presenting dysmorphic features, multiorgan dysfunction, severe hypoglycemia, and unexplained respiratory insufficiency [24]. Although there were no dysmorphic features suggesting particular syndromes in our case, the absence of anoxic events at birth, ASM-resistant seizures, and ensuing neuromuscular features such as hypotonia prompted us to perform a genetic evaluation in search of other underlying etiologies contributing to the atypical clinical course. We cannot explain these phenotypes by specific genes since the relationship between genotype and phenotype is still not well described in the 21q22.3 region. However, numerous pathogenic genetic conditions have been reported to be linked to HIE-like features as well as having increased susceptibility to hypoxic injury [8,25], such as genetic syndrome (e.g., Prader–Willi syndrome), inhered metabolic disorders, and genetic epileptic encephalopathies (*SIK1* gene) [25]. The neuropathogenesis of non-HI neonatal encephalopathy is also unclear. HIE-like manifestations could be explained by the increased susceptibility toward hypoxic brain injury. Genetic abnormalities associated with the activation of cytokines, increased neuroinflammation, mitochondrial dysfunction, and activation of neuronal apoptosis [26] have been reported to be predisposing conditions toward brain hypoxia, irrespective of obvious hypoxic insults. 

Overall, non-HI neonatal encephalopathy resulting from a genetic cause could be easily overlooked and often lead to diagnostic confusion because of subtle differences in its clinical presentation [23]. Our case highlights the importance of distinguishing HIE from non-HI neonatal encephalopathies by carefully considering the current history and their clinical course to determine appropriate standardized care such as therapeutic hypothermia. Even with a high index of suspicion of HIE, newborns with dysmorphic features, neonatal seizures, neuromuscular features or MRI findings of non-typical HIE should undergo genetic testing to identify a treatable inherited disorder. Likewise, in the case of neonates having clinical signs similar to HIE without obvious hypoxic insult in the perinatal period, we should delve further into possible secondary causes. Moreover, given the rarity of this chromosomal abnormality, more studies are needed to clarify certain genotype–phenotype correlations and minimize the candidate regions to provide the basis for precision diagnosis, gene-targeted therapy, and future genetic counseling.

In conclusion, we present a newborn with de novo 21q deletion in ring chromosome 21 who initially manifested HIE-like variables that could easily lead to misdiagnosis. This case contributes to the further clinical recognition of this rare 21q deletion in ring chromosome 21 as well as emphasizing the importance of multidisciplinary approach and clinical follow-up to disclose other underlying etiologies causing non-HI neonatal encephalopathy. Furthermore, advanced technology in investigating genomics such as bridging clinical phenotype–genotype in chromosome 21 and identifying putative pathogenic genes in the 21q region is expected to play a critical role in the near future. 

## Data Availability

The data presented in this study are available upon request from the corresponding author. The data are not publicly available due to privacy restrictions.
